# The Introduction of Myo-Inositol in the Synthesis of Rigid Polyurethane-Polyisocyanurate (RPU/PIR) Foams and Its Effect on RPU/PIR Properties

**DOI:** 10.3390/polym17222986

**Published:** 2025-11-10

**Authors:** Joanna Liszkowska, Krzysztof Moraczewski

**Affiliations:** 1 Department of Chemistry and Technology of Polyurethanes, Faculty of Materials Engineering, Kazimierz Wielki University, J.K. Chodkiewicza 30, PL 85-064 Bydgoszcz, Poland; 2 Department of Polymer Materials Engineering, Faculty of Materials Engineering, Kazimierz Wielki University, J.K. Chodkiewicza 30, PL 85-064 Bydgoszcz, Poland; kmm@ukw.edu.pl

**Keywords:** inositol, polyurethane foam, antioxidant, polymers, degradation, aging, polyphenols, polyisocyanurate foam

## Abstract

Myo-inositol (cis-1,2,3,5-trans-4,6-cyclohexanehexol) (In) was incorporated into rigid polyurethane/polyisocyanurate (PU/PIR) foams to investigate its effect on the degradation and performance properties of the foam, as well as its structure. The parameters studied included production temperature, processing times, strength, absorbency, and flammability. The foams were aged (degraded) in a special degradation chamber. The test results indicated the effect of myo-inositol on the foam properties. The addition of In caused a reduction in the cell diameter of the foams (measured in both directions). Absorptivity and water absorption decreased. The compressive strength of the foams increased and the flammability decreased (increased retention and decreased burning rate). As a result of foam degradation, the thickness of the degraded foam layer containing 13 wt.% myo-inositol (In13_D) increased by approximately 30% compared to the reference foam (In0_D).

## 1. Introduction

A number of compounds are known in medicine to reduce cell aging [1]. The effect of bioactive senolytic and senomorphic polyphenols (resveratrol, kaempferol, apigenin, and fisetin) on aging-related processes was described by Vedova et al. [2]. Antioxidants have been obtained, among others, from peppermint, from bilberry, grape, pomegranate, from ginkgo biloba, pink pepper, from wheat and rye [3,4,5], tomato fruit, apples, aronia melanocarpa [6,7,8,9], goji berries, acai berry, white mulberry, phaseolus vulgaris, cocoa, coffee, cinnamon, and mango seed kernel extracts [10,11]. Research has also been conducted on antioxidants in red wine, which contains anthocyanins and flavonols [12,13,14,15,16,17]. Antioxidant activity in argan, almond, sesame, nigella, sunflower, and soybean press cake has also been conducted [18]. Sunflower press cake outperformed in total phenolic and flavonoid content.

One well-known antioxidant is inositol. Often referred to as vitamin B8 (although technically not a vitamin), it is an organic compound belonging to the polyol group, which plays a key role in numerous biological processes. It is naturally present in the body and in plant and animal products such as citrus fruits, whole grains, nuts, and legumes. In the body, inositol acts as a cell signal transmitter and participates in lipid metabolism and nervous system function. One important aspect of inositol’s action is its role as an antioxidant. Thanks to its ability to neutralize free radicals, inositol helps protect cells from oxidative stress, which is one of the main causes of aging and the development of neurodegenerative, cancer, and metabolic diseases. Inositol and its derivatives, such as phosphatidylinositol and myo-inositol, have the ability to regulate oxidative processes by:

- Reducing the level of reactive oxygen species (ROS)—helping to limit cellular damage caused by oxidative stress,
- Supporting mitochondrial function—improving cellular energy efficiency, which is important for brain and nervous system health,
- Influencing glucose and lipid metabolism—supporting insulin function, which is important in the prevention of type 2 diabetes and polycystic ovary syndrome (PCOS).
- Due to its antioxidant properties, inositol is increasingly used as a dietary supplement to support the treatment of various conditions such as: neurological and mental disorders (depression, anxiety disorders, bipolar disorder), metabolic diseases, including insulin resistance and polycystic ovary syndrome (PCOS), and cardiovascular diseases by regulating cholesterol and triglyceride levels [19,20,21].

Although primarily known for its role in biology and medicine, inositol is also used in the chemical industry, including the production of plastics and biodegradable polymers. Its unique structure and properties make it a valuable raw material in modern materials technologies.

**Figure 1:**
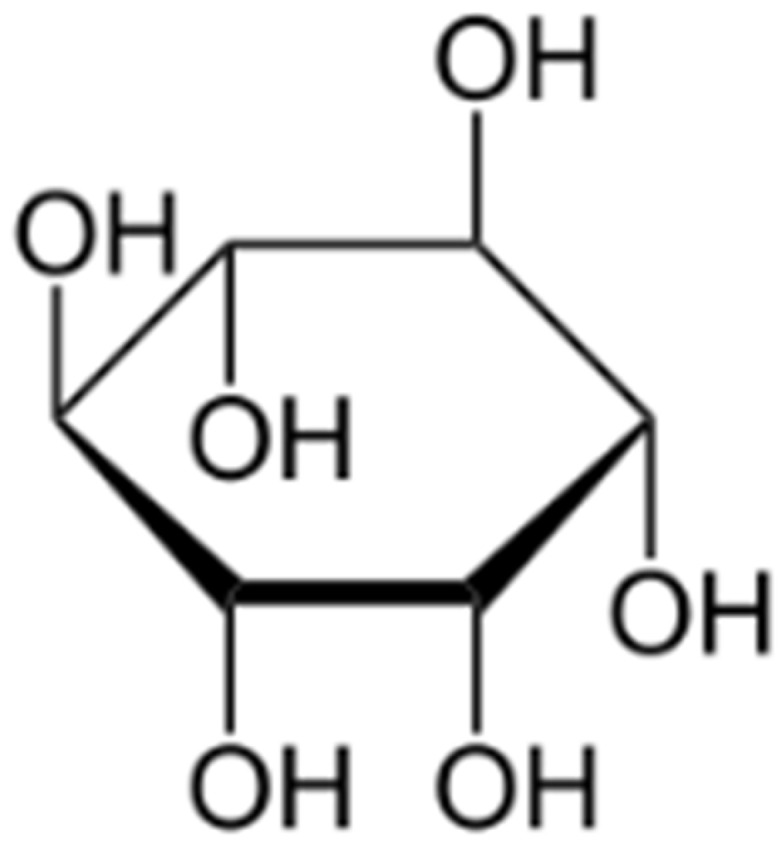
Myo-inositol—structural pattern.

Due to its cyclic structure and the presence of hydroxyl (-OH) groups (Figure 1), myo-inositol (cis-1,2,3,5-trans-4,6-cyclohexanehexol) can be used as a substrate for the synthesis of biodegradable polymers. It is a particularly promising compound in the production of: aliphatic polyesters, which are used as biodegradable alternatives to traditional plastics; polyurethanes with reduced toxicity, used in biomaterials and protective coatings; and polymer hydrogels, which are used in medicine, tissue engineering and drug delivery systems.

Inositol can also be used as an additive to polymers, improving their physicochemical properties. Thanks to its hydrogen bonding capacity and compatibility with many polymer systems, it can increase the flexibility, thermal stability, and biodegradability of materials. Some studies suggest that inositol esters can improve the hydrophilicity and biocompatibility of polymers, making them more environmentally friendly and suitable for medical applications. Thanks to their chemical reactivity, inositol and its derivatives can be used to create intelligent polymer coatings, such as moisture-absorbing and environmentally responsive self-healing materials, and biocompatible protective layers used in electronics and medicine [22,23,24].

There is little information in the literature regarding the use of inositol in polyurethanes. Studies highlight the versatility of myo-inositol as a bio-based monomer in polyurethane synthesis. An example of a publication on improving the properties of polyurethane is the use of myo-inositol, a naturally occurring cyclic polyol, into polyurethane synthesis to enhance the material’s properties [25]. The polyurethanes exhibited comparable thermal degradation behaviors. They also showed lower Tg (glass transition temperature) values compared to previously tested foams [26,27]. A notable study by Sudo and Kaiba [28] developed a method to synthesize hydroxyl-bearing polyurethanes using myo-inositol. The process involved bis-acetalization of myo-inositol with 1,1-dimethoxycyclohexane to produce a diol. This diol was then reacted with 1,3-bis(isocyanatomethyl)cyclohexane, a rigid diisocyanate, resulting in a polyurethane with a high glass transition temperature (Tg) of 192 °C. Subsequent treatment with trifluoroacetic acid hydrolyzed the acetal groups, yielding hydroxyl-functionalized polyurethanes. The presence of multiple hydroxyl groups facilitated hydrogen bonding, maintaining a high Tg of 186 °C. These hydroxyl groups also allowed for further side-chain modifications through reactions with isocyanates. 

Further research demonstrated the use of myo-inositol-derived polyurethanes as matrices for embedding chiral fluorophores. These composites exhibited solid-state circularly polarized luminescence, indicating potential applications in advanced optical materials [29].

Currently, the synthesis of polyurethanes (PU) is seeking alternative, harmless raw materials for their production [30]. Food waste from the oil industry is used for this purpose [18], which represents a valuable and profitable reservoir of potentially functional or bioactive compounds. The waste management policy is linked to the European Green Deal, which calls for a 55% reduction in greenhouse gas emissions by 2030 and the decarbonization of the EU economy by 2050, in line with the commitments of the Paris Agreement [31,32,33,34].

Currently, PUF polyurethane foams are made from toxic chemicals. PUF recycling is also challenging and is rarely utilized. Therefore, it is important to develop foam formulations using environmentally friendly compounds while meeting customer requirements regarding the properties of this two-phase material, in which the gas phase is to ensure a low heat transfer coefficient and the solid phase—high mechanical strength [30]. These conditions are met by PU fillers in the form of various types of waste, e.g., cellulose, waste, or coffee husks, wheat hulls, and rice hulls [35,36,37,38,39,40].

However, direct use of waste is not always possible. When using, for example, rapeseed or rice straw in rigid polyurethane foams, the isocyanate index should be lowered. Otherwise, the product’s stability decreases and the foams collapse [41]. Waste from the paper industry (waste paper, used packaging, cardboard) [42,43] and eggshells [44,45] have also been used in foams. These wastes are used directly in foams or after combustion, using the resulting ash [10]. However, the direct use of waste paper involves a number of processes, e.g., cleaning the paper from dyes or other substances contained in it.

In recent years, foams produced from vegetable oils have become popular, intended to replace petrochemical polyols [46,47,48]. This is also one of the ways to achieve sustainability in PUR synthesis [49]. For example, polyesterols from lipids, polyetherols from polysaccharides, and aromatic polyols from lignocellulose have been obtained [50,51,52]. Foams obtained from these bio-based polyols exhibited physical and mechanical properties comparable to those of PUR foams synthesized from petrochemical-based polyols [53,54,55,56,57,58,59]. Polyols were obtained from rapeseed oil, soybean oil, castor oil, palm oil [60,61], palm oil reinforced with cellulose nanocrystals, castor oil reinforced with carrot nanofibers, and lignin-based [62,63,64,65]. Polyesterols were also synthesized from citric acid [66,67,68]. This acid, in combination with glycols, produced polyesterol products, which were blended in various ratios with petrochemical polyols. This method of modifying the properties of rigid polyurethane-polyisocyanurate foams resulted in the creation of cheaper products with improved properties (e.g., strength).

The aim of the research in this article was to investigate the effect of myo-inositol (In) on the properties of rigid polyurethane-polyisocyanurate (PU/PIR) foams (In0–In13 foam). A three-week accelerated aging (degradation) process was carried out on these foams in a degradation chamber. This resulted in degraded foams (In0_D–In13_D). FTIR analysis was used to assess the type of chemical bonds present in the tested polymer. It was determined whether the peak intensity of individual bonds changed. Thermal tests were conducted to demonstrate the effect of the In filler on the thermal changes occurring in the foams. Flammability tests were conducted to determine the effect of In on the flammability of the foams. The structure of the non-degraded (non-aged) and aged foams was assessed.

## 2. Materials

To obtain a product with the appropriate properties (including stability, strength, and absorbency), it is necessary to prepare appropriate polyurethane (PU) formulations [69]. To achieve this, substrate A (polyol/diol with all components except B) is combined with substrate B (isocyanate) in equal amounts, provided that the foaming agent is not water. However, if foaming is performed with carbon dioxide, which is produced using water, the amount of isocyanate must be increased by the amount that will react with the water. During foaming, amines or urea are also formed, which react with the -NCO groups in the isocyanate [70,71]. Water present in the raw materials and additives must also be taken into account.

The formulation of the reference (In0) rigid polyurethane-polyisocyanurate (PU/PIR) foam obtained here contained: Rokopol RF 551 (79.2 g, PCC Rokita S.A., Brzeg Dolny, Poland), silicone Genapol X080 (5.6 g, Evonik, Essen, Germany), catalysts: Dabco (Alfa Aesar, Haverhill, MA, USA) in diethylene glycol (3.3 g, Chempur, Poland) and potassium acetate (Chempur, Poland) in diethylene glycol (8.2 g, Chempur, Poland), flame retardant Roflam F5 (49.4 g), water (3.15 g), and isocyanate Purocyn B (250.7 g, Purinova, Bydgoszcz, Poland). All data on the raw materials are contained in the aforementioned article [72]. The composition of the In0 foam was modified by the addition of powdered myo-inositol In (bulk.com, Gunfleet, Colchester, C049QX, United Kingdom/Bulk Centrum Logistyczne, Lakowa street 23, 55-095 Mirków, Poland). Myo-inositol was supposed to be a white powder containing 100% myo-inositol. The manufacturer did not provide its density. The amounts of In powder added were 1 wt%, 3 wt%, 7 wt%, and 13 wt%, which were 3.18 g (foam In1), 9.54 g (In3), 22.26 g (In7), and 41.54 g (In13), respectively. Due to its chemical structure, it is planned to synthesize a new bio-polyol based on myo-inositol in order to replace petrochemical polyols for polyurethane foams and to investigate its effect on the properties of the polymer.

## 3. Methods

The foams were obtained using the block method according to [10,72,73]. The foam ingredients (except the isocyanate) were mixed using a 20 cm mechanical stirrer at 1800 rpm until completely mixed. After mixing the components of this polyol premix, the isocyanate was added and mixed for an additional 10 s. The mixture was poured into an open, rectangular mold measuring 190 mm × 190 mm × 230 mm. After preparation, the foams were thermostated at 100 °C for 6 h and then stored for 24 h at room temperature. The resulting foam blocks were cut into test samples with an accuracy of 0.1 mm and weighed with an accuracy of 0.01 g. The test methods are described in [10,72,73]. Among other things, the following were tested: the foams were analyzed for color, density, absorbency and water absorption, compressive strength, structure, FTIR, thermal properties (DSC, DTA), flammability and degradation.

The apparent density was measured on five 50 mm cube samples from each series (ISO 845:2006 [74]). The apparent density was the ratio of foam mass to its geometric volume.

The compressive strength was measured on five cubic samples (from each series) with a side length of 50 ± 1 mm (ISO 844:2021 [75]) using the Instron 5544 universal testing machine. The samples were subjected to 10% compressive strength in line with the direction of growth.

A microscope VHX-X1 (Keyence Corporation, Osaka, Japanese) was used to take images of the foam structure and measure the cell counts. The studies were performed at 250× and 500× magnification.

Infrared spectra obtained using a Nicolet iS10 FTIR (Thermo Fisher Scientific, Waltham, MA, USA) spectrometer with a DTGS detector allowed for the evaluation of the PU/PIR chemical structure. The spectroscopic range was 4000–400 cm^−1^, maximum resolution < 0.4 cm^−1^.

The flammability test was conducted using a simplified stack test (vertical Butler test) according to ASTM D3014 [76]. The apparatus consisted of a vertical column measuring 300 × 57 × 54 mm with a movable glass front. The test was performed on six samples measuring 150 × 19 × 19 mm. Before combustion, the samples were weighed to the nearest 0.001 mm and then placed in the stack. A propane–butane flame was applied for 10 s. The burner was removed, and the samples were reweighed. The sample’s combustion residue (retention) was measured as the ratio of the sample mass before combustion to the sample mass after combustion. The result was multiplied by 100%. Flammability was also measured using the vertical test with the PN-78 C-05012 (PN-C-05012-12:1978) standard [77]. Samples measuring 150 × 50 × 13 mm were placed on a horizontal standardized grid and exposed to a flame (propane–butane burner) from one end for 60 s. The surface flame spread rate was determined on the foam sample.

Absorbability (N) and water absorption (Ch) were determined in accordance with ISO 280 2896:2001 [78]. Water absorption of rigid cellular plastics, measured after immersion in distilled water for 24 h of foam samples with dimensions of 150 × 150 × 25 (mm).

TGA (thermogravimetric analysis) tests were carried out using a Q500 thermobalance (TA Instruments, New Castle, DE, USA) in a nitrogen atmosphere (temperature range from 0 to 1000 °C, temperature change rate 10 °C/min). The sample weighed approximately 21 mg. Based on the TG curves, the temperatures of 5% (T5), 20% (T20), and 50% (T50) loss of the initial mass of the sample were determined. From the differential DTG curve (first derivative of the TG curve) the T_max_ values (the temperature of the fastest mass loss) were also determined.

The examination of changes occurring in foams under heat was conducted using differential scanning calorimeter DSC Q200 (TA Instruments, New Castle, DE, USA) with built-in Advanced Tzero technology. The apparatus working range is from −90 to +725 °C (ashes examinations were conducted in the range from 0 to 400 °C) in nitrogen.

Resistance to aging under the influence of atmospheric factors plays an important role in foam applications. A test was conducted involving the controlled action of destructive factors, elevated temperature of 50 °C, relative humidity of 70%, and UV radiation of 320.86 W/m^2^. The aging test was conducted for 3 weeks in accordance with the requirements for polymers. Samples were placed in a heated climatic chamber (DYCOMETAL CCK, model CCK-40/300 NG, Es-tor Sp. z o.o., Poznań, Poland). The degraded foam surface was examined (color, structure, depth of the degraded layer), and FTIR, DSC, and DTA were performed.

**Figure 2:**
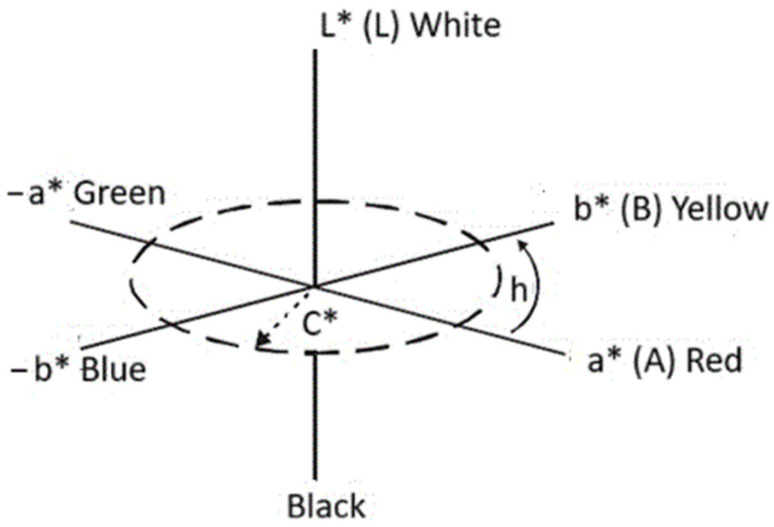
CIELAB color solid diagram (L*a*b*) and L* C* h.

Color measurements were performed using a BYK colorimeter (BYK-Gardner GmbH, Geretsried, Germany), designed for measuring the color and gloss of polymer materials. The SF80 Spectrophotomether (manufacturer: TRI-COLOR Sp. z o.o. Siedziba: ul. Jodlowa 50, 32-095 Narama, woj. małopolskie, Poland) [10,72,73] based on the CIE LAB model was used to determine the effect of the filler on the color of the foams. Developed by Hunter R.S. [79] it is based on three values L*, a*, and b* [80] contained in a three-dimensional color space (Figure 2). The vertical L* axis is lightness and can take values from 0 to 100. Positive values on the a* axis determine the amount of red, negative values the amount of green. The b* axis expresses the amount of yellow (positive values) or blue (negative values). The CIE L*a*b* coordinates can be converted to cylindrical coordinates L*, C*, and h using appropriate equations (Munsell color space). The L* value also determines lightness, and C* expresses color saturation and increases along the radius of the circle. The h value (tone angle) determines the shade of the color and is measured on a circle from the a* axis [81].

## 4. Results and Discussion

### 4.1. Foam Production

PU foam synthesis consists of the following stages: latent, growth, stabilization (gelation), and foam maturation [13]. The first stage involves increasing the volume of the mixture. The second stage involves exothermic polymerization and the formation of a cell structure. The third stage involves internal and side reactions, including the formation of allophanate and biuret bonds. The final stage can last up to several hours. All reactions are completed, and the final cell structure, shape, and size of the foam are determined. Each stage takes a specific time—Table 1.

Generally, cream time depends on the type and amount of catalysts used [70,71]. However, increasing the modifier content in the foams also had an impact, extending the start time from 10 s (In0, foam without myo-inositol) to 16 s (In13, foam with 13 wt.% of myo-inositol)—Table 1. However, string time and tack free time remained virtually unchanged with increasing myo-inositol (In) content, ranging from 27 s to 31 s for all foams. The free rise time for foam In0 was 40 s, and for the rest of the foam it was 30 s. The maximum temperature during the exothermic reactions decreased with increasing amounts of inositol in the foam, from 177 °C (In1) to 171 °C (In13), and for standard foam (In0) it was also 171 °C. The increase in the start-up time was influenced by the increasing density of the polyol masterbatch due to the addition of myo-inositol.

### 4.2. Color

**Figure 3:**
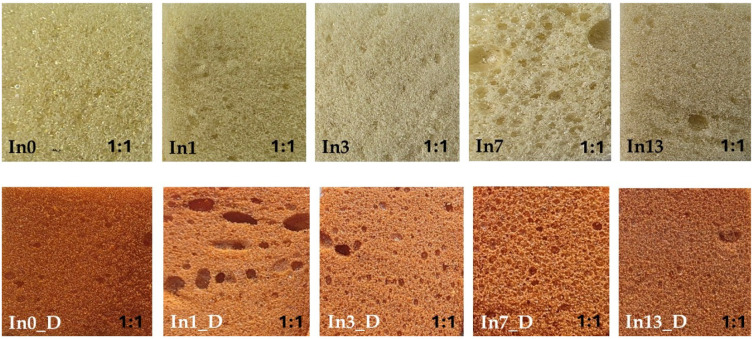
Foam color before degradation (In0–In13) and after degradation (In0_D–In13_D).

The results of the macroscopic color tests of the foams: In0–In13 (before degradation) and In0_D–In13_D (after degradation) are presented in Figure 3.

The spectrophotometric color test results for the foams before and after degradation are presented in Table 2. For the foams before degradation (In0–In13), as the inositol content in the foams increased, the color “warmed” slightly, i.e., it increased toward the red a* color (thereby decreasing toward green), while simultaneously increasing toward the yellow b* color (thereby decreasing toward blue). The foams also became slightly lighter (the L value increased). The value of the index defining the difference between two colors in the CIELab color space (ΔE) decreased from 80.79 (In1) to 75.09 (In13).

The degraded foams (In0_D–In13_D) were observed to become redder (the a* value increased approximately 20-fold). A darkening of the degraded samples was also observed (the L value, responsible for brightness, decreased by approximately 60%) compared to the undegraded foams (In0–In13). The color change was caused by factors including the UV light present in the degradation chamber. Chromophores in PUR foams produced from aromatic isocyanates interact with light. However, this process does not lead to a deterioration of the material’s physical properties [82,83]. Measurements showed that the difference in ΔL values between the undegraded and degraded foams was approximately 21–24.

### 4.3. FTIR

**Figure 4:**
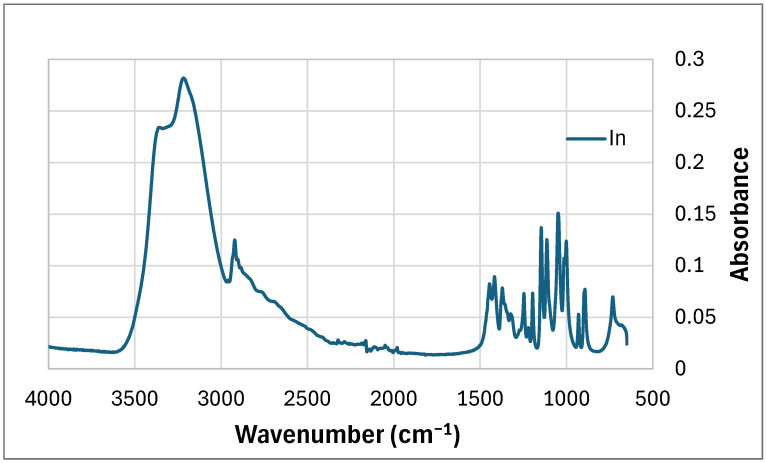
FTIR of mya-inositol (In).

The main peaks observed in the FTIR graph of inositol (Figure 4) are the peaks at wavenumber: corresponding to -OH groups (3358 cm^−1^), -CH of aromatic compound (3220 cm^−1^), -CH band (2922 cm^−1^), two peaks representing -OH groups derived from phenols (1445 cm^−1^ and 1400 cm^−1^), bands derived from -C-OH groups (from 1250 cm^−1^ to 1001 cm^−1^) and C-C bonds in the region of 1300 cm^−1^–800 cm^−1^.

**Figure 5:**
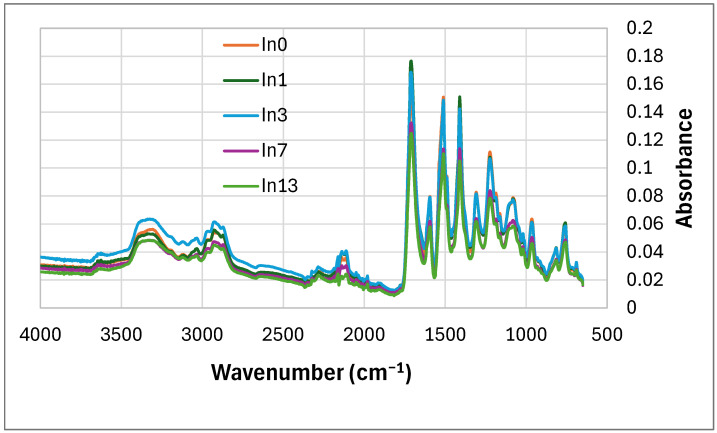
FTIR of foams before degradation: reference foam In0 and foams with In (In1–In13).

The FTIR results of the non-degraded foams are shown in Figure 5. The foam contains a urethane (-NHCOO-) and isocyanurate bond. This bond is attributed to the peaks of functional groups in the molecular structure, such as: C–O–C asymmetric stretching (1075–1081 cm^−1^), -CN stretching (1224 cm^−1^), N-H bending (1511 cm^−1^, 950 cm^−1^, 758 cm^−1^), C=O stretching (1713 cm^−1^) and N–H stretching vibrations derived from amines (3340 cm^−1^) [84], tiny anti-symmetric stretching vibration of the unreacted -N=C=O groups coming from isocyanate (2300 cm^−1^) [85] indicating the reaction of isocyanate and the formation of an isocyanurate ring (large peak at 1411 cm^−1^) [86]. Two stretching bands -CH were also observed at 2969 cm^−1^ (derived from -CH_2_-) and at 2867 cm^−1^ [87]. The bands occurring in the region of 1660–1740 cm^−1^ are attributed to carbonyl groups C=O, present, for example, in ester, urethane, allophanate, urea derivatives, and isocyanurate groups [88].

With the increase in inositol in the foams (from In0 to In13), the type of bands did not change. The intensity (absorbance) of the first eight peaks increased slightly, depending on the content of mya-inositol (In) from 0 wt.% (In0) to 3 wt.% (In3). Then, this peak intensity decreased (from In3 foam to In13 foam).-Table 3. Starting from the peak at wavenumber 1308 cm^−1^ peak intensity decreased with increasing In content in the foam.

**Figure 6:**
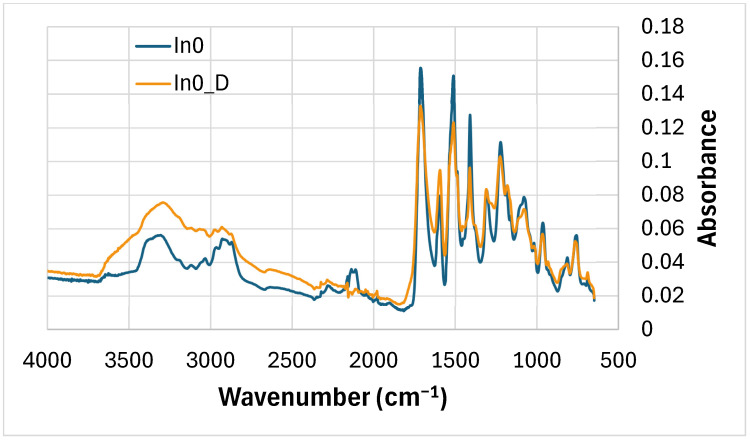
FTIR comparison: In0 foam (before degradation) and In0_D foam (after degradation).

The absorbance of the peak of the degraded reference foam (without myo-inositol) In0_D at wavenumbers around 3340 cm^−1^ (-NH) increased compared to the absorbance of this peak for the undegraded In0 foam—Figure 6, Table 3. However, the absorbance of the peaks around 2100 cm^−1^ and 2150 cm^−1^, as well as at 1600 cm^−1^, decreased. The remaining peaks also decreased in absorbance after degradation, e.g., around 1530 cm^−1^, 1411 cm^−1^ (isocyanurate ring), 1220 cm^−1^, 1035 cm^−1^ and 950 cm^−1^ (-NH) [85,87,89,90,91].

**Figure 7:**
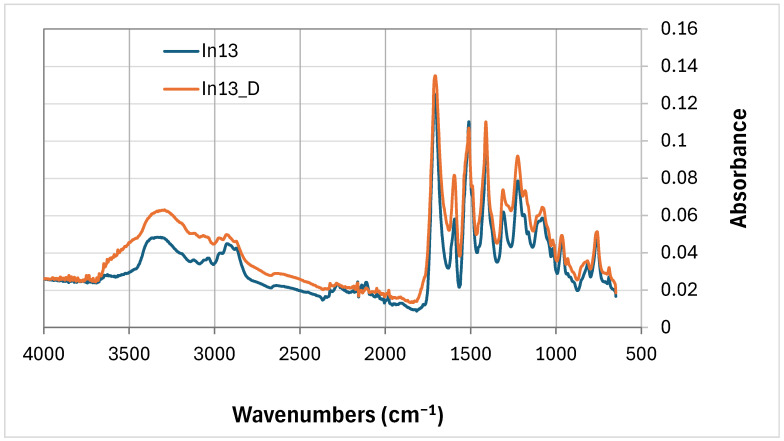
FTIR comparison of 13 wt.% mya-inositol containing foam not degraded (In13) with degraded foam (In13_D).

Comparing the FTIR curves of the foams with 13 wt% myo-inositol (In13 and In13_D), the FTIR line pattern (Figure 7) is inversely related to the comparison. In0 and In0_D foams (Figure 6). The absorbance of the peak at wavenumbers around 3340 cm^−1^ decreases for the degraded foam (In13_D). At 2100 cm^−1^ the absorbance of the peak (In13) due to degradation (In13_D) decreases and at wavenumber 2050 cm^−1^ the absorbance of all peaks of the degraded foam In13_D increases (except 1490 cm^−1^).

### 4.4. Foam Structure

Foam structure is formed by the formation of gas bubbles in the polymer matrix [92,93]. Pore size is particularly important for the final material properties. Cell anisotropy (height-to-length ratio) influences the physical properties of foams [10,40,72].

**Figure 8:**
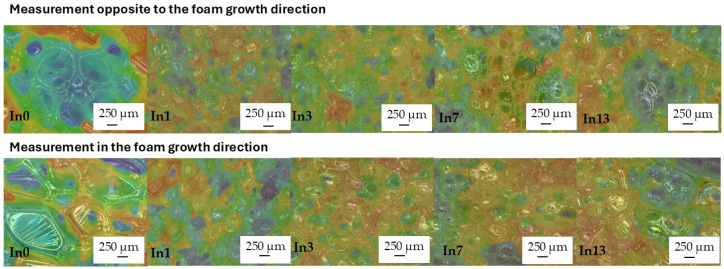
Study of cell anisotropy before degradation.

After adding mya-inositol to the reference foam In0, the cells of all foams decreased approximately three-fold (Figure 8) and became rounded. Inositol could act as a nucleator, promoting the formation of smaller and more uniform cells. Inositol improved the anisotropy of the undegraded foams tested in the opposite direction (op) to the foam growth direction from 1.16 μm (In0_op) to 1.08 μm (In13_op). This was due to slower foam processing parameters due to the increased density of the polymer masterbatch (see Table 1). Similarly, the anisotropy of the foams measured opposite to the growth direction (op) improved. The elongated cells of the In0 foam (measured opposite to the foam growth) also became rounded after the addition of inositol (In1_in–In13_in foams)—Table 4. This was beneficial for the increase in compressive strength.

**Figure 9:**
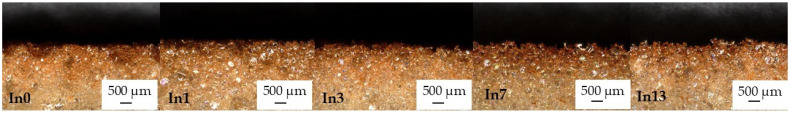
Measurement of the thickness of the degraded layer in In0_D–In13_D foams.

The thickness of the degraded foam layer (Figure 9) changed with increasing inositol content, from 1441 μm (In0) to 2023 μm (In13)—Table 4.

**Figure 10:**
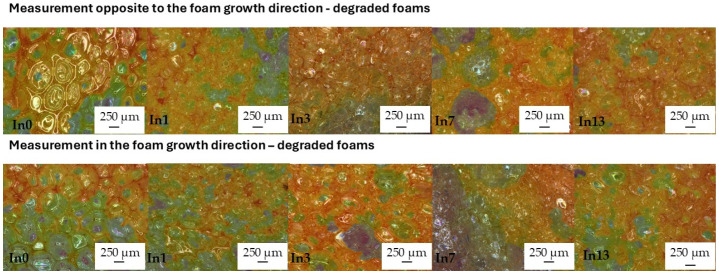
Study of cell anisotropy after degradation.

As a result of foam degradation, no shrinkage was observed, and the images show no wrinkles or creases in the cell walls (In0_D–In13_D)—Figure 10.

Cell diameter measurements of the degraded foams showed an approximately 30% increase in the diameter of the degraded cells compared to the cells of the non-degraded foams—Table 4. This was caused by loosening of the structure by moisture, radiation, and temperature. PUR and PIR foams contain ester bonds (especially PUR) as well as urethane and isocyanurate bonds (in PIR), which are sensitive to water—especially in the form of steam and elevated temperatures. Hydrolysis of the ester and urethane bonds ultimately leads to their cracking. Byproducts such as alcohols and carboxylic acids are formed, which can further react or increase the polarity of the material. This weakens the cell wall structure. The cell walls soften or crack, which can increase their size. UV radiation leads to the breakdown of polymer chains (photooxidation). Free radicals are generated, which cause further chain breakdown. The polymer’s molecular weight decreases, and the material loses its cohesiveness. UV can also degrade foaming agents and flame retardants. This leads to brittleness, weakening of the cell walls, deformation, or stretching. Elevated temperature can accelerate both hydrolysis and oxidation processes. Temperature softens the polymer and increases the gas pressure within the enclosed cells. As a result, the cell walls can expand or crack. In the long term, this leads to a loss of dimensional stability, delamination, and changes in cell size. Degradation by these three factors simultaneously may have resulted in a partial collapse of the microstructure, resulting in a visible change in the dimensions of the foam cells [94,95,96,97,98,99,100,101,102,103]. Studies indicate that complete degradation did not occur, but rather softened and stretched the cell walls, particularly in the direction of foam growth. Mya-inositol likely protected the walls from complete disintegration. Only an increase in cell height and width occurred. The cells elongated primarily in the direction of foam growth (to a lesser extent in the direction of growth counterclockwise), which is why there was a slight increase in cell anisotropy from 1.12 μm (In0_D_in) to 1.75 μm (In13_D_in)—Table 4.

### 4.5. Britleness

**Figure 11:**
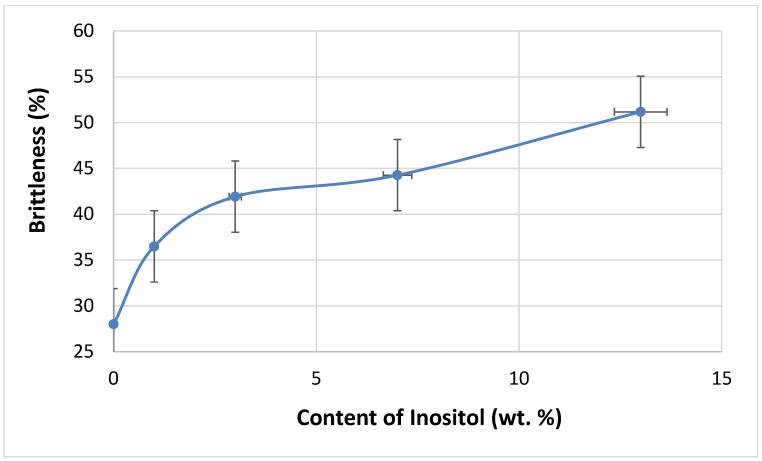
Dependence of inositol content in the foam on brittleness.

Increasing the filler content in the polyol masterbatch (component A) increased the viscosity of the reaction system, thus reducing the mixing efficiency of the components. As a result, some properties of the foams deteriorated, e.g., brittleness increased with increasing inositol content in the foams from 28.00% (In0) to 51.17% (In13)—Figure 11.

### 4.6. Absorptivity and Water Absorption

**Figure 12:**
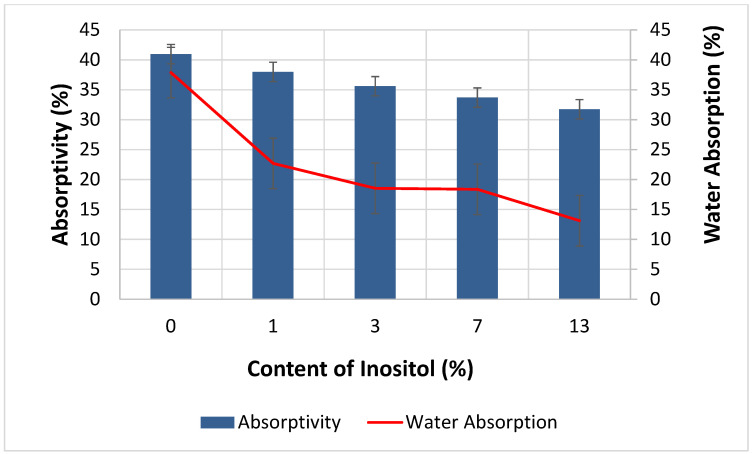
Dependence of Inositol Content in the Foam on Absorptivity and Water Absorption.

The addition of myo-inositol had a positive effect on the water absorption properties of the foams. Increasing the filler content in the foams simultaneously decreased absorbency from 40.96% (In0) to 31.75% (In13) and water absorption from 37.87% (In0) to 13.12% (In13)—Figure 12. Myo-inositol, as a filler, could influence foaming kinetics and the formation of cell structure. It could act as a nucleator (smaller and more uniform cells were formed). Smaller, regular cells remained closed more often, and closed cells were impermeable to water. An increased fraction of closed cells directly contributed to a decrease in water absorption. The introduction of myo-inositol molecules altered the rheology of the polyurethane mixture (longer processing times). Higher viscosity resulted in a decrease in the energy required to rupture the cell membranes, resulting in fewer open cells and, consequently, less water access and lower water absorption. Myo-inositol could partially integrate into the polyurethane network. This network became more compact (Section 4.4) and hydrophobic, which limited the penetration of water molecules. Small particles of myo-inositol could be deposited on cell walls or in interfacial spaces, creating a physical barrier to moisture penetration [96,100,104,105,106,107].

### 4.7. Density and Compressive Strength

**Figure 13:**
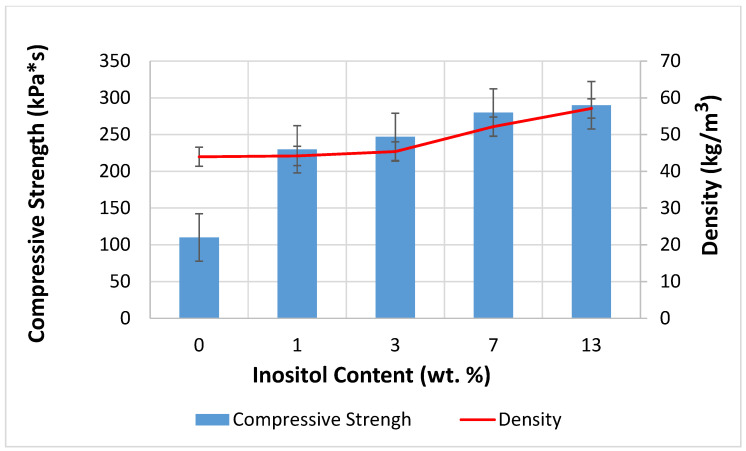
Dependence of Inositol Content in the Foam on Compressive Strength and Density.

The addition of myo-inositol to the foams increased the density from 43.97 kg/m^3^ to 57.12 kg/m^3^ and increased the compressive strength from 110 kPa*s to 290 kPa*s (Figure 13). Due to its crystalline structure, myo-inositol improved the foams’ strength. Incorporating myo-inositol into the foam structure increased the cross-link density. The material became stiffer and less susceptible to deformation. The formation of smaller, more uniform cells resulted in better stress dissipation. A higher cell wall-to-volume ratio promotes improved mechanical properties. Myo-inositol caused a reduction in foam expansion, making the foam more compact, which increased its density. Higher foam density almost always correlates with higher compressive strength because there is more material per unit volume. Inositol filled the interphase spaces, which limited the movement of chain segments and mechanically reinforced the polymer matrix [100,104,107,108,109,110].

### 4.8. Flammability

Polyurethane foams are chemically inert and non-toxic. However, as flammable materials, they can pose a fire hazard. Burning foams produce large amounts of carbon monoxide, hydrogen cyanide, nitrogen oxides, and other toxic gases. This motivates PU manufacturers to modify them to reduce flammability [71,83,111]. Phosphorus, halide, nitrogen, and clay compounds are primarily used [112].

**Figure 14:**
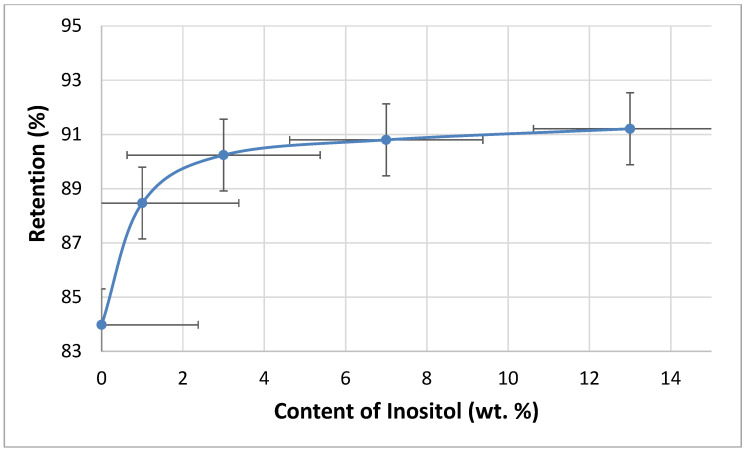
Dependence of inositol content in the foam on retention.

**Figure 15:**
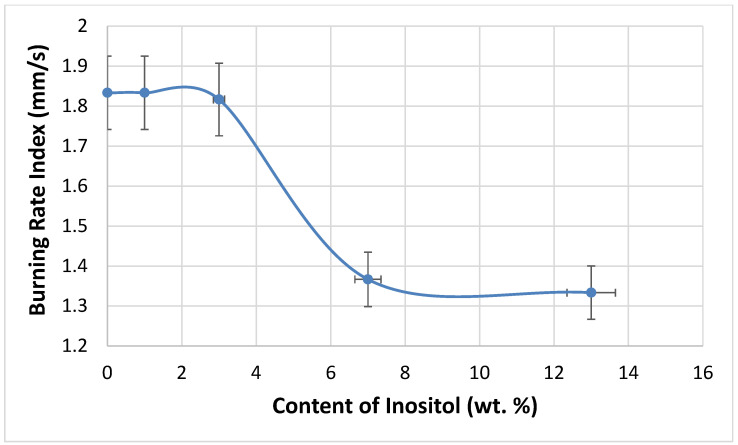
Dependence of inositol content in the foam on the burning rate index.

The flammability of the foams was tested using the vertical Butler stack test (Figure 14) and the horizontal stack test (Figure 15). The combustion residue (retention) of the foams increased with the inositol content from 83.98% (In0) to 90.80% (In13). Meanwhile, the foam’s burning rate decreased from 110 mm/s (In0 and In1) to 80 mm/s (In13).

Inositol therefore contributed to the increased fire resistance of the foams. Probably by sealing the foam structure (see Section 4.4), it caused the cells to close. This contributed to the formation of an insulating layer and reduced air access to the foam interior. Additionally, the ring-shaped myo-inositol structure reduced the foam’s flammability. Myo-inositol can act as a physical filler that prevents fire from spreading. It reduces the effective amount of flammable organic phase, even if it is not chemically modified. During combustion, it forms solid residues (ash/char) that protect the foam surface. In addition, the reduced cells in the structure limit the flow of smouldering liquids to the deeper layers of the foam, which delays the initiation of combustion and hinders the spread of flames [97,113,114].

### 4.9. TGA

**Figure 16:**
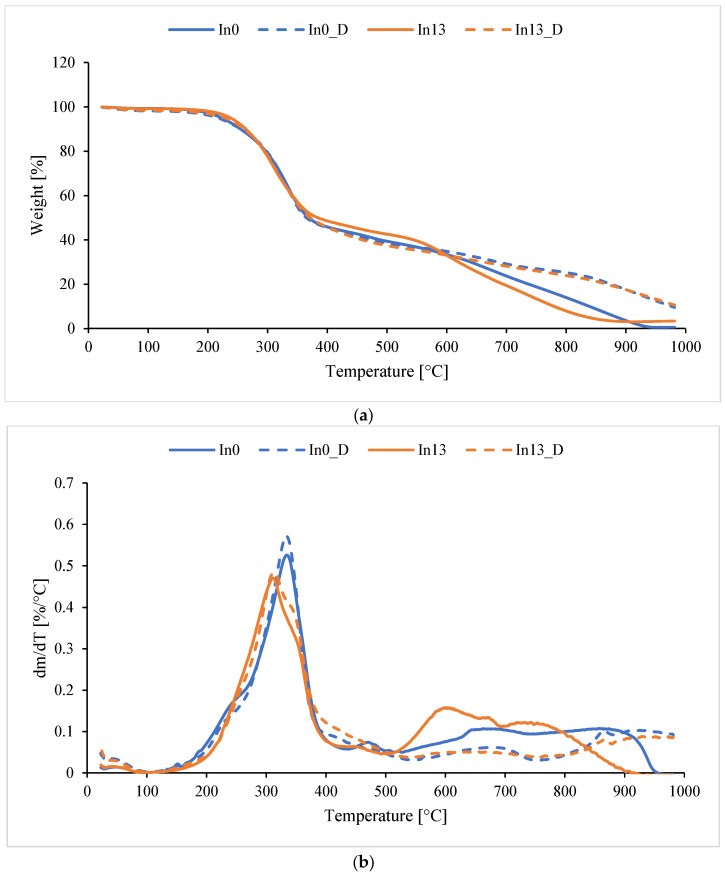
TGA of foams: (**a**) TG, (**b**) DTG.

Using TG and DTG, the temperatures at which 5%, 10%, 20%, and 50% mass loss occurred in the non-aged In0–In13 and aged In0_D–In13_D foams were determined. The temperature of maximum mass loss Tmax was also determined at each stage of foam decomposition. A sample TGA graph (inositol-free foam and with the highest inositol content, aged and non-aged) is presented in Figure 16 (a—TG, b—DTG). The thermal test results are included in Table 5 and Table 6.

Thermal stability at 5% mass loss of non-degraded foams (In0–In13) ranges from 311 to 340 °C—Table 5. For aged foams (In0_D–In13_D), the T5% value decreases to 217 to 229 °C compared to non-degraded foams. The T5% value increases with increasing myo-inositol content in the foam from 217.6 °C to 229.3 °C, which indicates the ability of inositol to delay the initial decomposition temperature of the foam. This increase may be due to increasing myo-inositol content. For residue at 1000 °C, no dependence of residue on inositol was observed for either degraded or non-degraded foams. Overall, it can be seen that the modified samples (especially In1 and In13) have significantly higher decomposition temperatures than the reference sample (In0). After degradation (samples D), some foams gain in stability (e.g., In1_D, In3_D—very high T50%). Sample In1_D leaves the highest residue (22.5 mg), which may indicate the formation of stable residues after degradation. Modification with inositol significantly increases the amount of residues compared to In0.

Reading the data in Table 6 allowed for a better understanding of the foam degradation mechanism. Tmax1–Tmax4 (°C) is the temperature of the maximum mass loss rate in a given stage (from the DTG curve), while Δm1–Δm3 (%) is the percentage of mass lost in a given stage (main stages). Tmax4 represents the later, often slow, decomposition of residues.

In most cases, the foams underwent thermal decomposition in four stages (Table 6). The mass loss of the foams in stage 1 is quite significant and amounts to about 50% (Tmax1 around 330 °C). In stage 2, the mass loss is much smaller and amounts to 3.8–16.2% (with Tmax2 in the range of 456–507 °C), and in stage 3, the mass loss is 22.1–10.0% (Tmax3 in the range of 603.7–780.3). Tmax1 remained relatively stable (changes ±5 °C), except for In1 (+4.5 °C), which indicates a slight increase in resistance in the first stage. For Tmax2, small changes are observed, but a noticeable increase is observed in In7_D (+35 °C), suggesting stronger bonds of intermediate products after degradation. For Tmax3, a strong decrease was observed in In3_D (–145.4 °C), suggesting a faster decomposition of stable structures. In the case of Tmax4, an increase was observed in most cases, especially in In13_D (+201.5 °C), which may suggest the formation of more resistant residues. In the case of mass changes, Δm1 decreased in all samples (e.g., In1_D −7.8%, In3_D −17.3%), indicating that less degradation occurred in the first stage. This suggests possible degradation occurred earlier. Δm2 increased significantly, especially in In1_D (+12.7%) and In7_D (+8.5%). Here, the degradation shifted to the second stage. In the case of Δm3, the differences were variable: the largest decrease occurred in In13_D (–11.6%), and an increase occurred in In3_D (+0.7%). This may indicate that less material passed to the final degradation.

### 4.10. DSC

According to the literature [115], polymer hydrolysis begins at the surface, and the greater the polymer’s affinity for water, the faster the hydrolysis. Therefore, the introduction of isosorbide improves the affinity between the copolyester and water, allowing water to completely permeate the copolyester surface and improving its hydrolysis rate.

**Figure 17:**
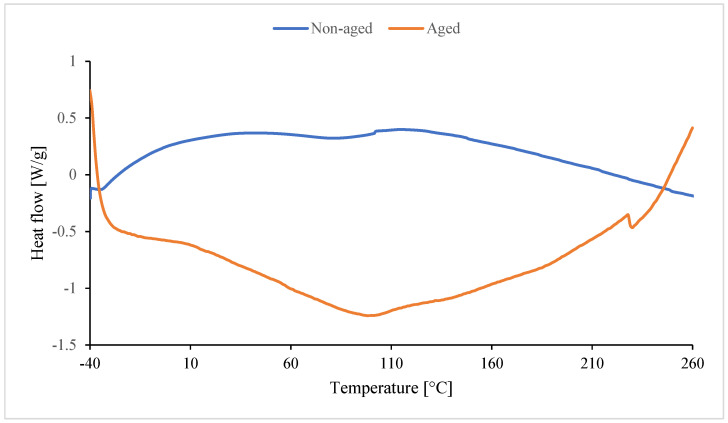
Result of DSC of reference foam without inositol (non-aged In0 and aged In0_D).

**Figure 18:**
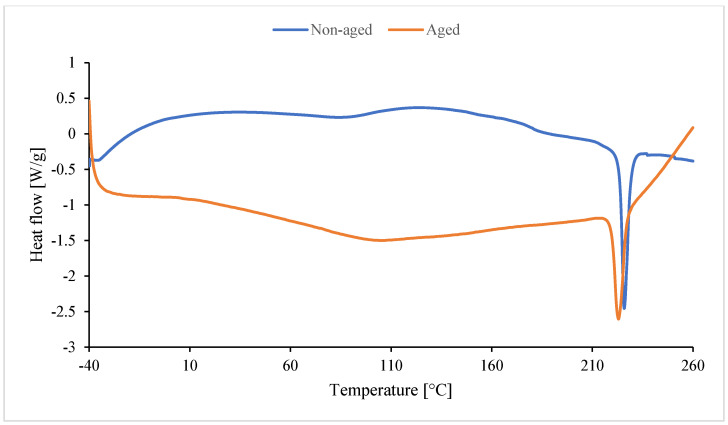
Result of DSC of foam with 13 wt.% of inositol (non-aged In13 and aged In13_D).

In the case of non-aged foam (blue line), the peak is upward. Unreacted substrates or byproducts formed during foam synthesis are likely reacting. They react during the DSC analysis and undergo cross-linking. Amines, for example, formed during the synthesis of isocyanate with water, can also react as a byproduct of CO_2_ formation. These amines also react with excess isocyanate to form urea [116]. Dimers and trimers are also formed. Cross-linking occurs as a result of the formation of allophanate and biuret bonds. The red peak (aged foam) may be the result of an endothermic reaction of water (high moisture content during degradation). The melting peak around 225–227 °C is absent in In0 foam (Figure 17) and occurs only in foams with inositol (Figure 18). This peak results from the melting of mya-inositol. The heat flow at this point increases with increasing In content in the foam.

Before degradation (Table 7), Texo1 and Texo2 probably correspond to the temperatures of exothermic effects associated with cross-linking or rearrangement reactions in the polyurethane structure. The introduction of inositol (In1–In13) changes the course of thermal effects, especially for Texo1 and Texo2. For low contents (In1–In3), an increase in Texo1 is observed (up to ca. 53 °C) compared to the In0 sample (39.9 °C). This indicates a more ordered structure of the soft segments and greater thermal stability. For higher contents (In7, In13), Texo1 decreases to ca. 22–23 °C. This suggests a decrease in the mobility of the soft segments, probably due to excess inositol, which may interfere with cross-linking or create its own hydrogen bonds. Δhexo1 decreases significantly for In7 (141 J/g), indicating a lower amount of energy associated with thermal transformations. This may indicate a less regular structure. A temperature of approximately 225–227 °C appears only in samples containing inositol, but not in In0. This suggests that inositol promotes the formation of more ordered hard segments (PIR) with a distinct melting point. The enthalpy of melting (Δhexo2) increases with the amount of inositol (from 2.5 to 28.1 J/g), confirming a greater proportion of the crystalline phase or ordered PIR domains.

After degradation (Table 8), all samples exhibit a decrease in the endothermic effect temperature (Tendo1). This indicates a weakening of the PUR/PIR structure due to the degradation of the soft segments. For the foams with inositol (especially In7_D and In13_D), the melting effect persists at approximately 223 °C (Tendo2), suggesting that the hard PIR phase with inositol is more resistant to degradation. ΔHendo2 increases with inositol content (from 1.8 J/g to 18.8 J/g), confirming a higher proportion of ordered PIR domains, even after degradation. The reference sample In0_D has the highest Tendo1 (145.7 °C), but the lack of a second peak indicates that it does not form stable crystalline domains like the samples with inositol.

## 5. Conclusions

Although the use of inositol in the plastics industry still requires further research and optimization of production processes, its potential as a component of biopolymers, additives for synthetic materials, and functional coatings makes it a valuable compound in modern materials science. The myio-inositol (In) used here has a beneficial effect on strength parameters, reducing foam flammability, absorptivity and water absorption. The addition of In slightly interferes with the foaming process, and processing times are only slightly extended. This fact should be taken into account when preparing foam formulations. However, it can be beneficial in various applications where extended processing parameters are necessary. Processing parameters can be controlled and adjusted using appropriately selected types and amounts of catalysts. The addition of mya-inositol reduces the flammability of RPU/PIR and increases the surface area after sample degradation. The addition of myo-inositol also affects the thermal properties of the foams. A small addition of inositol (In1–In3) improves thermal stability and the degree of structural order, while its excess (In7, In13) leads to partial disruption of foam cross-linking, despite the increase in the share of the rigid phase. Inositol improves the thermal stability of the rigid phase and the structure’s resistance to degradation, but in large quantities it can reduce the stability of the soft segments. After degradation, samples containing inositol still exhibit ordered domains (T_endo2_ ≈ 223 °C), indicating more stable isocyanurate (PIR) bonds.

## Figures and Tables

**Table 1:** Foam processing parameters.

Foam	Cream Time (s)	String Time (s)	Tack Free Time (s)	Free Rise Time (s)	T_max_ (°C)
In0	10	28	31	40	171
In1	13	26	28	30	177
In3	14	26	27	30	174
In7	15	27	29	30	172
In13	16	28	30	30	171

**Table 2:** Change in color of foams before (In0–In13) and after degradation (In0_D–In13_D).

Foam	L (-)	a* (-)	b* (-)	ΔE (-)
In0	63.51	−4.99	20.50	66.92
In1	72.61	−1.37	35.41	80.79
In3	70.53	−1.8	32.77	77.79
In7	67.07	−0.52	35.70	75.98
In13	66.91	−0.93	34.07	75.09
In0_D	42.25	18.17	31.94	55.99
In1_D	48.41	16.77	35.28	62.20
In3_D	48.14	18.04	33.62	61.43
In7_D	45.60	19.98	38.14	62.71
In13_D	45.50	19.02	34.02	59.91

**Table 3:** Absorbance selected peak intensity results: foam before degradation (In0–In13) and foam after 3 weeks of degradation (In0_3–In13_3).

Wavenumber (cm^−1^)	Absorbance Before Degradation (-)			Absorbance After Degradation (-)		
	In0	In3	In13	In0	In3	In13
3340	0.56	0.64	0.49	0.062	0.075	0.075
2965–2974	0.049	0.055	0.040	0.048	0.059	0.059
2867–2872	0.051	0.057	0.043	0.046	0.057	0.057
2285–2323	0.025	0.031	0.023	0.023	0.030	0.030
2138	0.036	0.041	0.023	0.022	0.025	0.020
2106–2111	0.037	0.041	0.024	0.024	0.026	0.021
1713 (1660–1740)	0.155	0.170	0.125	0.013	0.014	0.013
1595	0.080	0.079	0.058	0.093	0.095	0.081
1511	0.151	0.149	0.110	0.122	0.131	0.107
1411	0.129	0.142	0.104	0.096	0.131	0.110
1308	0.082	0.081	0.061	0.061	0.081	0.081
1224	0.112	0.106	0.079	0.011	0.106	0.078
1075–1081	0.079	0.079	0.059	0.079	0.078	0.058
950–964	0.063	0.061	0.046	0.061	0.060	0.045
758–765	0.056	0.058	0.046	0.057	0.058	0.046

**Table 4:** Microscopic analysis results.

Foam	Cell Hight (μm)	Cell Width (μm)	Anisotrophy (-)	Degraded Layer Thickness (μm)
In0_op	538	463	1.16	-
In1_op	256	249	1.03	-
In3_op	254	229	1.11	-
In7_op	257	230	1.12	-
In13_op	236	219	1.08	-
In0_in	747	410	1.82	-
In1_in	256	229	1.12	-
In3_in	255	230	1.11	-
In7_in	278	265	1.05	-
In13_in	257	230	1.12	-
In0_D_op	556	528	1.05	-
In1_D_op	254	234	1.08	-
In3_D_op	338	257	1.32	-
In7_D_op	217	198	1.10	-
In13_D_op	412	313	1.31	-
In0_D_in	462	413	1.12	1441
In1_D_in	355	314	1.13	1592
In3_D_in	403	315	1.28	1709
In7_D_in	503	345	1.50	1642
In13_D_in	452	288	1.75	2035

op—opposite to the foam growth direction, in—in the foam growth direction.

**Table 5:** TG and DTG before degradation (In0–In13) and after degradation (In1_D–In13_D).

Foam	T_5%_ (°C)	T_10%_ (°C)	T_20%_ (°C)	T_50%_ (°C)	Residue at 1000 °C (mg)
In0	223.4	254.8	296.9	368.7	0.6
In1	237.0	266.9	303.8	379.4	4.3
In3	235.2	265.2	300.7	362.2	13.0
In7	239.2	268.8	301.8	374.5	12.5
In13	238.1	263.6	294.3	384.2	3.4
In0_D	217.6	255.4	298.4	365.0	9.6
In1_D	227.6	267.6	307.9	398.8	22.5
In3_D	222.8	261.0	301.7	441.9	2.5
In7_D	226.0	261.1	299.8	387.3	10.0
In13_D	229.3	260.9	295.4	371.5	10.6

**Table 6:** Stages of foam decomposition in DTG and their temperature maxima.

Foam	Tmax1 (°C)	Δm1 (%)	Tmax2 (°C)	Δm2 (%)	Tmax3 (°C)	Δm3 (%)	Tmax4 (°C)
In0	334.8	55.7	470.6	5.6	662.9	18.0	857.2
In1	335.0	55.3	467.5	3.5	650.1	15.9	927.4
In3	335.1	61.6	480.7	4.4	780.3	20.5	-
In7	340.9	58.2	471.9	3.8	633.2	11.1	943.7
In13	311.2	51.5	455.0	5.1	603.7	22.1	769.3
In0_D	333.6	52.6	468.1	8.8	679.9	10.0	925.7
In1_D	339.5	47.5	468.0	16.2	-	-	918.6
In3_D	332.3	44.3	463.5	8.9	634.9	19.8	920.0
In7_D	334.3	49.5	507.0	12.3	673.9	13.7	854.2
In13_D	312.7	51.7	456.0	10.6	655.8	10.5	970.8

**Table 7:** Results DSC of foam.

Foam	Texo1 (°C)	Texo2 (°C)	Δhexo1 (J/g)	Tm (°C)	Δhexo2 (J/g)
In0	39.9	115.2	329.2	-	-
In1	51.5	115.4	352.9	226.0	2.5
In3	53.8	112.5	303.0	227.4	6.1
In7	22.4	147.0	141.1	225.7	12.9
In13	23.2	123.8	289.3	225.9	28.1

**Table 8:** Results DSC of foam (after degradation).

Foam	Tendo1 (°C)	ΔHendo1 (J/g)	Tendo2 (°C)	ΔHendo2 (J/g)
In0_D	145.7	98.0	-	-
In1_D	115.0	105.0	222.9	1.8
In3_D	107.8	81.9	222.9	5.0
In7_D	101.9	113.5	222.9	11.9
In13_D	105.9	93.0	222.9	18.8

## Data Availability

The original contributions presented in this study are included in the article. Further inquiries can be directed to the corresponding author.

## Abbreviations

The following abbreviations are used in this manuscript:

In	mya-inositol
In0–In13	non-aged foams
In0_D–In13_D	aged foams
Dataset	
Dataset License	
